# Motor Training Using Mental Workload (MWL) With an Assistive Soft Exoskeleton System: A Functional Near-Infrared Spectroscopy (fNIRS) Study for Brain–Machine Interface (BMI)

**DOI:** 10.3389/fnbot.2021.605751

**Published:** 2021-03-18

**Authors:** Umer Asgher, Muhammad Jawad Khan, Muhammad Hamza Asif Nizami, Khurram Khalil, Riaz Ahmad, Yasar Ayaz, Noman Naseer

**Affiliations:** ^1^School of Mechanical and Manufacturing Engineering (SMME), National University of Sciences and Technology (NUST), Islamabad, Pakistan; ^2^Florida State University College of Engineering, Florida A&M University, Tallahassee, FL, United States; ^3^Directorate of Quality Assurance and International Collaboration, National University of Sciences and Technology (NUST), Islamabad, Pakistan; ^4^National Center of Artificial Intelligence (NCAI), National University of Sciences and Technology, Islamabad, Pakistan; ^5^Department of Mechatronics and Biomedical Engineering, Air University, Islamabad, Pakistan

**Keywords:** brain machine interface (BMI), brain computer interface (BCI), machine learning (ML), mental workload (MWL), functional near infrared spectroscopy (fNIRS), exoskeleton, bionic actuating behavior, neuroergonomics

## Abstract

Mental workload is a neuroergonomic human factor, which is widely used in planning a system's safety and areas like brain–machine interface (BMI), neurofeedback, and assistive technologies. Robotic prosthetics methodologies are employed for assisting hemiplegic patients in performing routine activities. Assistive technologies' design and operation are required to have an easy interface with the brain with fewer protocols, in an attempt to optimize mobility and autonomy. The possible answer to these design questions may lie in neuroergonomics coupled with BMI systems. In this study, two human factors are addressed: designing a lightweight wearable robotic exoskeleton hand that is used to assist the potential stroke patients with an integrated portable brain interface using mental workload (MWL) signals acquired with portable functional near-infrared spectroscopy (fNIRS) system. The system may generate command signals for operating a wearable robotic exoskeleton hand using two-state MWL signals. The fNIRS system is used to record optical signals in the form of change in concentration of oxy and deoxygenated hemoglobin (HbO and HbR) from the pre-frontal cortex (PFC) region of the brain. Fifteen participants participated in this study and were given hand-grasping tasks. Two-state MWL signals acquired from the PFC region of the participant's brain are segregated using machine learning classifier—support vector machines (SVM) to utilize in operating a robotic exoskeleton hand. The maximum classification accuracy is 91.31%, using a combination of mean-slope features with an average information transfer rate (ITR) of 1.43. These results show the feasibility of a two-state MWL (fNIRS-based) robotic exoskeleton hand (BMI system) for hemiplegic patients assisting in the physical grasping tasks.

## Introduction

Tetraplegia and stroke are among the major causes leading to lesser control over muscular movements (Blokland et al., 2014). Patients suffering from such diseases show a declining trend in the uncontrolled motor movements during the later stages of the disease. These patients cannot control their motor movements due to neuronal degeneration (Lo et al., 2016; Hong et al., 2018). In the severe phase of these diseases, a patient may become completely paralyzed and unable to perform daily routine tasks. Spinal cord injuries (SCI) as well as some brain injuries may contribute to motor disabilities. The pattern of neural and hemodynamic signals in patients with the brain and spinal injuries also differs from that of healthy patients (Käthner et al., 2017). For such patients, there is a need to devise a methodology to partially, if not fully, rehabilitate them, helping them in performing routine tasks (Hong and Santosa, 2016). The brain–computer interface (BCI) and brain–machine interface (BMI) are among such neurofeedback methods that can provide rehabilitation and assistance to patients with severe motor disabilities (Khan and Hong, 2017). A BCI translates the neuronal hemodynamic signals acquired directly from a patient's brain into useful machine commands that can be used to control devices for assistance (Hong et al., 2020). Based on portability, low cost, and non-invasiveness, techniques like electroencephalography (EEG) and functional near-infrared spectroscopy (fNIRS) are commonly used in rehabilitation (Hong and Khan, 2017).

In comparison, fNIRS has a superior spatial resolution, while EEG has a better temporal resolution (Ferrari and Quaresima, 2012; Asgher et al., 2020b). BCI-based applications are now gaining significance and becoming more practical. A BMI system mainly comprises of four essential parts: signal processing, feature extraction, classification, and command generation (Asgher et al., 2019). Among these parts, signal processing and feature extraction are vital and applied in further utilizing neurofeedback systems. Over the years, EEG was used as a default for BCI applications. Recently, fNIRS is becoming prevalent to utilize a person's cognitive states for BCI and BMI applications (Naseer and Hong, 2015). Hemodynamic behaviors and responses of a healthy person are different from those of the patients. The flow of blood is also not the same for a stroke patient or brain injury victim compared with a healthy person (Kübler and Birbaumer, 2008; Chodobski et al., 2011; Kaufmann et al., 2013; Käthner et al., 2017).

One of the trailed paradigms in BMI is to detect the abstract body kinematics using neuro-imaging modalities and decode them using the regression model, and mapping them with social robotics. Abiri et al. (2017) presented a work in which the scalp EEG was recorded, and the user was visualizing different body kinematics. The studies (Volosyak, 2011; Ortiz-Rosario and Adeli, 2013) gives an overview of non-invasive EEG signals' processing techniques for SSVEP-based applications and similarly (Hong et al., 2018) presents a comprehensive study of different useful features in fNIRS-EEG-based activities. Likewise, Naseer and Hong (2015) and Zhang et al. (2019) discussed different machine and deep learning techniques used in fNIRS and EEG for hybrid BCI applications. Erkan and Akbaba (2018) described that minimum energy combination (MEC) and canonical correlation analysis (CCA) could be used in the detection of SSVEP signals during EEG recording, and MEC is recommended for synchronous SSVEP stimulus. Gao et al. (2003) showed the feasibility of SSVEP using an electric apparatus. In this study (Gao et al., 2003), the patient is introduced to different flickering lights (boxes), which are flashed at different rates and represents different actions against each (chosen from a menu). In various other studies, fNIRS signals are recorded to measure emotions and cognitive processing from the PFC region (Asgher et al., 2018, 2019). Some studies employed fNIRS to detect motor imagery and mental arithmetic tasks (Thanh Hai et al., 2013; Naseer and Hong, 2015).

It is essential to have a brain signal acquisition mechanism to ensure proper control for BCI and BMI. Along with brain signals, a prober haptic and prosthetic system is required for the patient to perform routines tasks. Research studies mostly focus on the BMI techniques' design while ignoring the neuroergonomic and human–machine interaction's (HMI) design factors like design parameters of the haptic device and appropriate integration with BMI. The role of EEG-based assistive mobile robots for patients with disabilities are identified in various studies (Brose et al., 2010; Diez et al., 2013). In these studies, researchers presented different applications of assistive BMI. Among the applications of mobile robots, the wheelchair is the most prominent one. Recently, some studies have emphasized more on the medical ergonomic aspects of the patients. Liu et al. (2016) and Chen et al. (2018) presented control of a seven-degree-of-freedom (DOF) robotic arm using 15 different possible choices. In these studies, the maximum number of commands per minute is 15, with an accuracy of 92%. The patients who suffered from tetraplegia (those unable to move their upper limbs) are studied (Pfurtscheller et al., 2008). They integrated EEG with functional electrical stimulation (FES) ascertained that integrated signals work better than EMG signals with the applied frequencies of 12, 15, and 20 Hz, and the average accuracy achieved was 70%.

High-speed BCI spellers are also essential to give the facility of communication to the people, who cannot speak irrespective of the cause. Diez et al. (2013) and Nakanishi et al. (2014) presented a high-speed BCI speller with a frequency resolution of 0.2 Hz and simulation time from 2 to 3 s. Meng et al. (2016) showed another technique similar to that of Zhang et al. (2017), where the subject was “pick and place” the object using a three-DOF robotic arm. The Intention-Driven Semi-autonomous Intelligent Robotic (ID-SIR) system is discussed in the study (Zhang et al., 2017), which is designed for assistive drinking tasks using P300 BMI and operation time of 82 s. For obtaining an object's boundary, the region-growing algorithm is applied by Khan et al. (2014), which gave a hybrid NIRS-EEG-based control with four different commands and Khan and Hong (2017) with eight different commands for quadcopter's control. In Asgher et al. (2019), the authors classify two-state mental workloads (MWL) from subjects using fNIRS, and the signals can be further utilized in neurofeedback. Rea et al. (2014) used fNIRS signals to detect lower limb movement for gait rehabilitation in chronic stroke patients with an acquired accuracy of 67.77 ± 11.35%. In Khan et al. (2018), the authors presented an fNIRS-based neurorobotic interface for gait rehabilitation. S. Perry, in his opinion article (Perrey, 2014), discussed fNIRS-based neural gait control to relevant cortical areas.

Various studies investigate the use of fNIRS as an objective guage of MWL and its serviceability testing and applied MWL-BCI in ecological environments (Karran et al., 2019). In a BCI system, the central nervous system (CNS) activities are measured and converted into output that enhances and improves CNS output by changing the interactions among the CNS and external environmental factors (Wolpaw and Wolpaw, 2012). In fact, BCIs may not necessitate or require any deliberate muscle's control, and dependent relative on brain's hemodynamic and motor response, so the choice of using BCI system mainly relies on the patient's adaptableness, ergonomics and so BCI roles and assists as a viaduct to get sensory input into the brain. Therefore, BCI systems are mostly designed as user specific and according to the abilities and capabilities of patients in a specific environment (Tariq et al., 2018). The BCI system's accurateness is also effected and decreased owing to the lack of patient's ability to hold and retain similar cognitive mental states in various experimental trials conducted in few studies as it is ascertained during these studies that that long periods of procedure may introduce mental cognitive fatigue for the patients (Papanastasiou et al., 2020). The applications of BCI systems are modest in clinical and medical environments owing to various factors like the BCI accuracy, BCI reliability of sensory system interface and control translation algorithms that encompasses constraints like time, ITR, the number of optodes or electrodes, and the number of distinguished emotions (Al-Nafjan et al., 2017). This study tries to address these limitations by utilizing cognitive load acquisition with fNIRS for BMI system. The present study gauges its operational effectiveness neuroergonomic factor MWL in BMI settings, and the purpose was to engage the cognitive load in operational environment, where normal motor or encoded open and close commands signals are not always possible especially under stress conditions. fNIRS-BMI studies on soft exoskeleton control are very limited (Lalitharatne et al., 2013; Gao et al., 2016; He et al., 2018). The proposed study utilizes fNIRS signals in operational BMI and tries to explain that fNIRS-based exoskeleton can be a potential research area for BMI, and comparison is performed with mostly existing literature on BCI-EEG studies.

The prospects of BCI system applicability beyond the laboratories by designing applications in ecological applications is defined as passive brain–computer interface (pBCI) (Aricò et al., 2018). In pBCI he system identifies the impulsive and spontaneous brain's response related to the cognitive states (emotional state, mental workload, stress, vigilance, fatigue, attention level), and utilizes this kind of data to enhance and regulate the interface among persons and the environment or system. Thus applied pBCIs are designed to meet the requirements of the system w.r.t (Zander and Jatzev, 2012). In this study, the task selection is also considered specific to evoke a functional response of potential patient's brain and type of activity suitable for the potential patient as well as healthy participant. In case of a stroke patient, operational commands were tried to be generated, the neural blood flow and pathways of the patients are different in case of the injury or disease (Birbaumer and Cohen, 2007; Chodobski et al., 2011; Käthner et al., 2017). The reported accuracy in the case of patients is less compared with that in healthy patients owing to the differences in neural and hemodynamic patterns either due to disease or injury (Burns et al., 2014; Costa et al., 2016; Käthner et al., 2017; Rieke et al., 2020). The MA tasks are widely used in literature to engage a certain amount of MWL (Schudlo et al., 2013; Kosti et al., 2018; Asgher et al., 2019). MWL is proposed as a specific task, so that even under stress and cognitive load, the patients could generate control command signals to operate the exoskeleton, and the study gauge its operational effectiveness. Moreover, MWL commands may be efficiently gauged with NASA TLX (Mansikka et al., 2019; Mingardi et al., 2020). This study assessed cognitive load using National Aeronautics and Space Administration's Task Load Index (NASA TLX), a multi-dimensional assessment method with six sub-scales: including mental demands, performance, effort, and frustration (Felton et al., 2012). fNIRS system measures and application of machine and deep learning classification algorithms able to discriminate between cognitive states even in cases of verbal or spatial tasks. Various studies investigate that verbalization also do not affect the fNIRS measurements as artifacts and fNIRS provide a strong indication of the participants cognitive load (MWL), and that may be utilized to ascertain the MWL objectively during various cognitive tasks (Maior et al., 2015). Using a combination of NASA-TLX and fNIRS methodology, task validation was performed.

**Figure 1:**
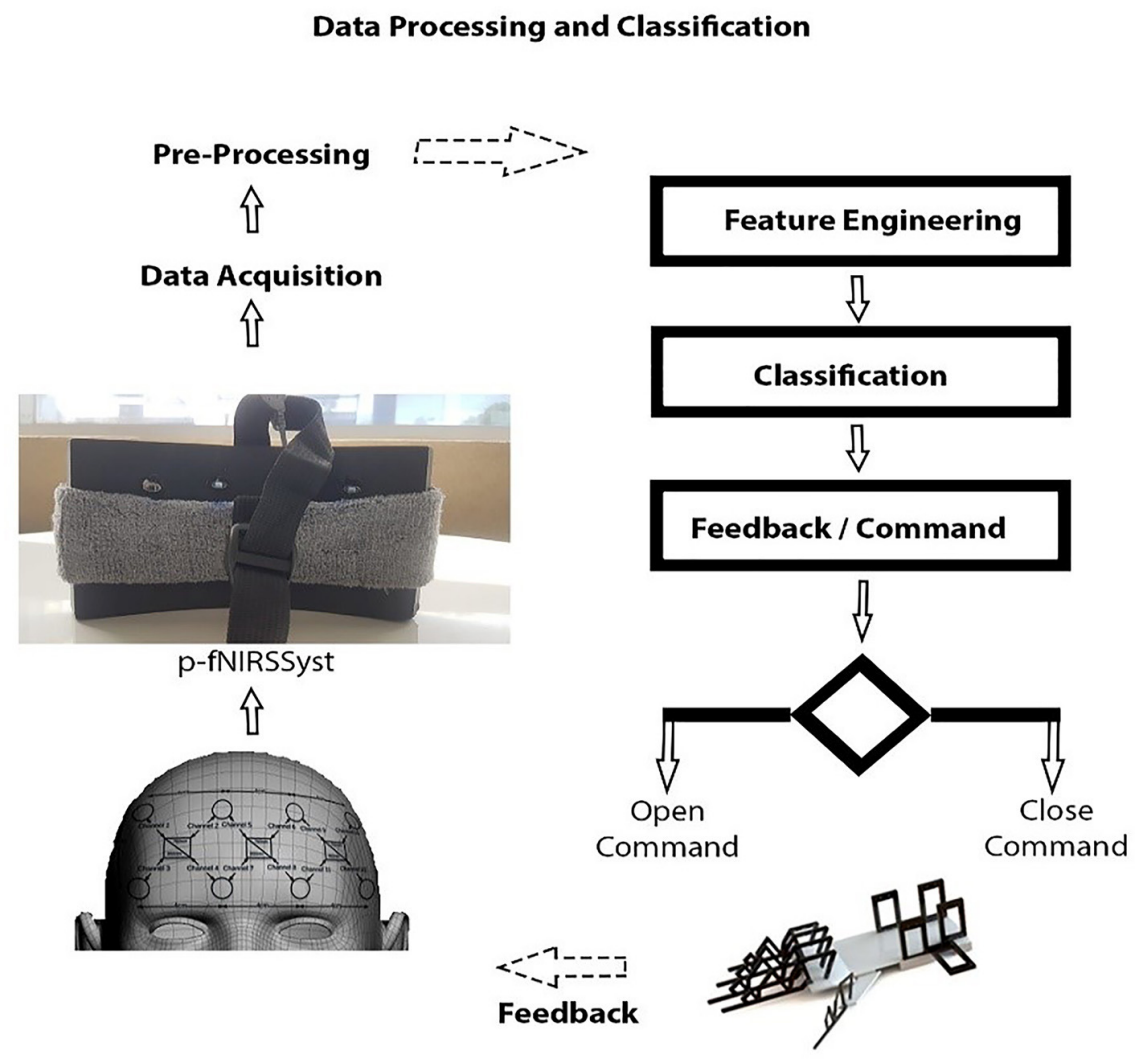
Mental workload (MWL) command-based brain–machine interface (BMI) system.

In this research, the neuroergonomic aspects of MWL are taken into consideration while designing the data acquisition fNIRS system from the brain (PFC) as well as the translation of brain signals to soft lightweight wearable robotic exoskeleton hand. Here in this study, a conceptual BMI designed is proposed with a soft exoskeleton hand. A novel fNIRS-based lightweight wearable exoskeleton hand mechanism for potential hemiplegic patients (performing daily routine tasks) is presented. Unlike previous studies (Ramadan and Vasilakos, 2017; Wang et al., 2019), the designed wearable exoskeleton has separately controllable five fingers as an added HMI factor with improved accuracy. A 12-channel fNIRS system is used for data acquisition recording (Asgher et al., 2019). The system acquired fNIRS signals and measured the two-state MWL. Data of 14 subjects with a mean age of 23.7 years participated in this study were utilized in classification and analysis. Two optimal feature combinations from the hemodynamic (HbO and HbR) signals, namely, mean and slope, were extracted and employed using a support vector machine (SVM) classifier. The maximum accuracy is 91.31%, with the average accuracy of 87.9%. The complete BMI system is summarized in Figure 1.

## Materials and Methods

### Experimentation

#### Methodology

**Figure 2:**
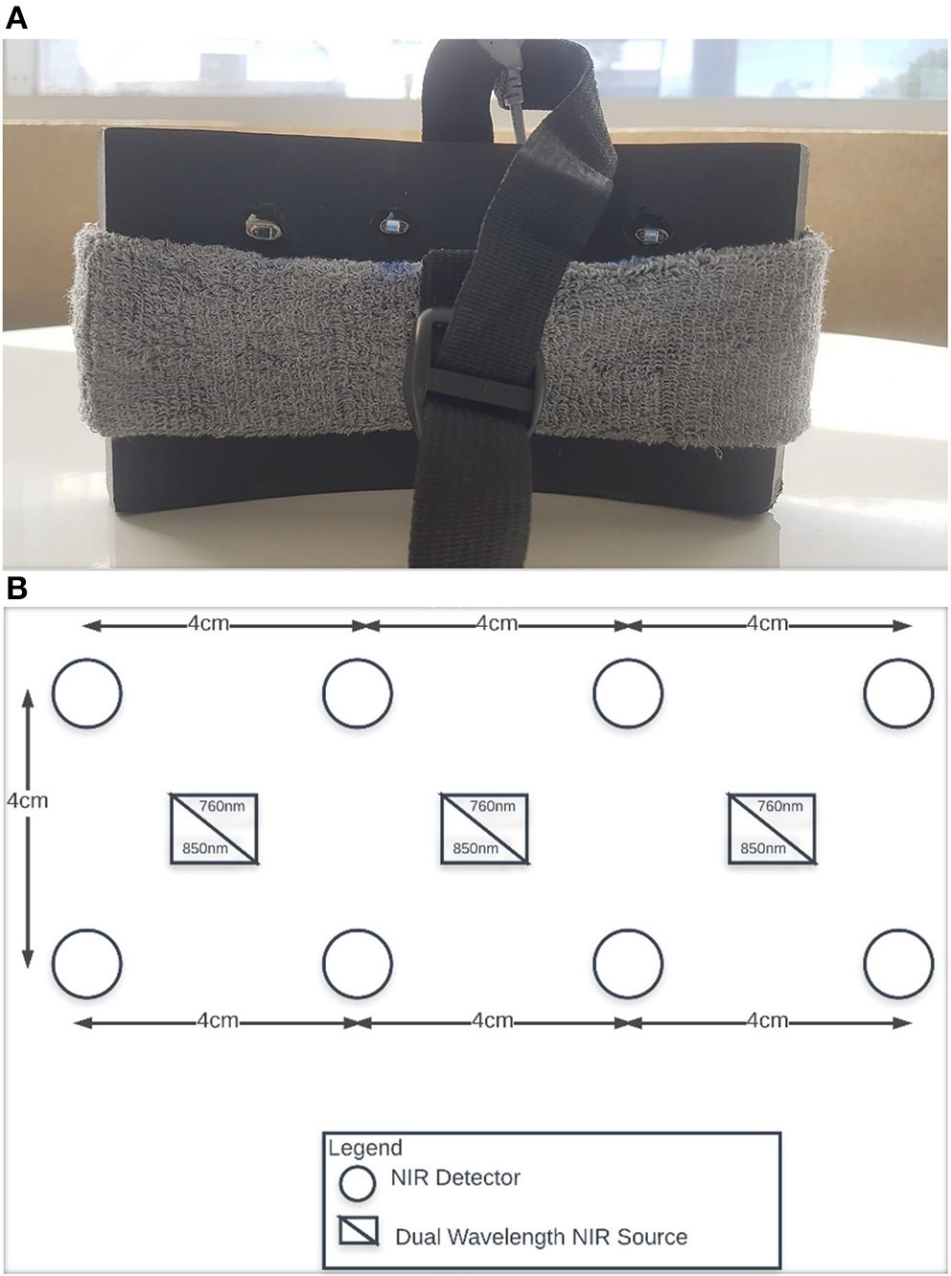
**(A)** Compact data acquisition fNIRS system (P-fNIRSSyst) with optodes placement and head-band. **(B)** Twelve channels of P-fNIRSSyst system with three sources and eight detectors.

The proposed architecture consists of two different parts. First is an experimental setup based on MWL assessment generated during the cognitive load assessed with fNIRS. The second is a wearable robotic exoskeleton hand. In this study, 12 channels with a two-wavelength continuous wave (CW) fNIRS “P-fNIRSSyst” system (Asgher et al., 2019) is employed to compute neuronal activation in the form of hemodynamic concentration changes in the brain. The obtained changes in concentration, intensity values of hemoglobin, are transformed into relative concentration changes of ΔHbO and ΔHbR using the modified Beer–Lambert law (MBLL). Samples are attained at 8 Hz sampling rate. The placement of fNIRS optical optodes is shown in Figure 2. Figure 2A is the neuroergonomic headband designed for easy use on the human head's PFC, and Figure 2B shows the placement of sources and detectors. The rectangles represent the sources, and the circle represents the detector. The distance between the source and detector is a channel, and there are 12 channels in the fNIRS system (Asgher et al., 2020a).

#### Participants

A total of 16 subjects (11 males and 5 females) initially participated in this study with age ranging from 20 to 27 years, mean age of 23.5 years, and standard deviation of 5.5 years. All the participants were engineering graduates. Medical screening of participants was performed under the medical doctor's supervision to assess any physical, neurological, or psychological issues or disease. None of the participants had any disability. They were given the details about the experiment before the start of the experimentation (task, time, and number of trials for MWL). The fNIRS recording of one subject (16th) was more than 10% contaminated with channel noise and so that specific subject's data were excluded from further analysis. The remaining 15 subjects' (10 males and 5 females) data were then analyzed and classified. After data cleaning, bad channel rejection, and data modeling, the final data of 14 subjects were used in the final analysis. Pre-assessment was conducted in the form of an interview to gauge their mathematical and analytical skills with control conditions like similar educational background and experience. The experimentation procedure was conducted under the Declaration of Helsinki and approved by the Ethical Research Council of RISE lab at SMME-National University of Sciences and Technology (NUST).

### Hardware: Design Optimization of Robotic Exoskeleton Hand

**Figure 3:**
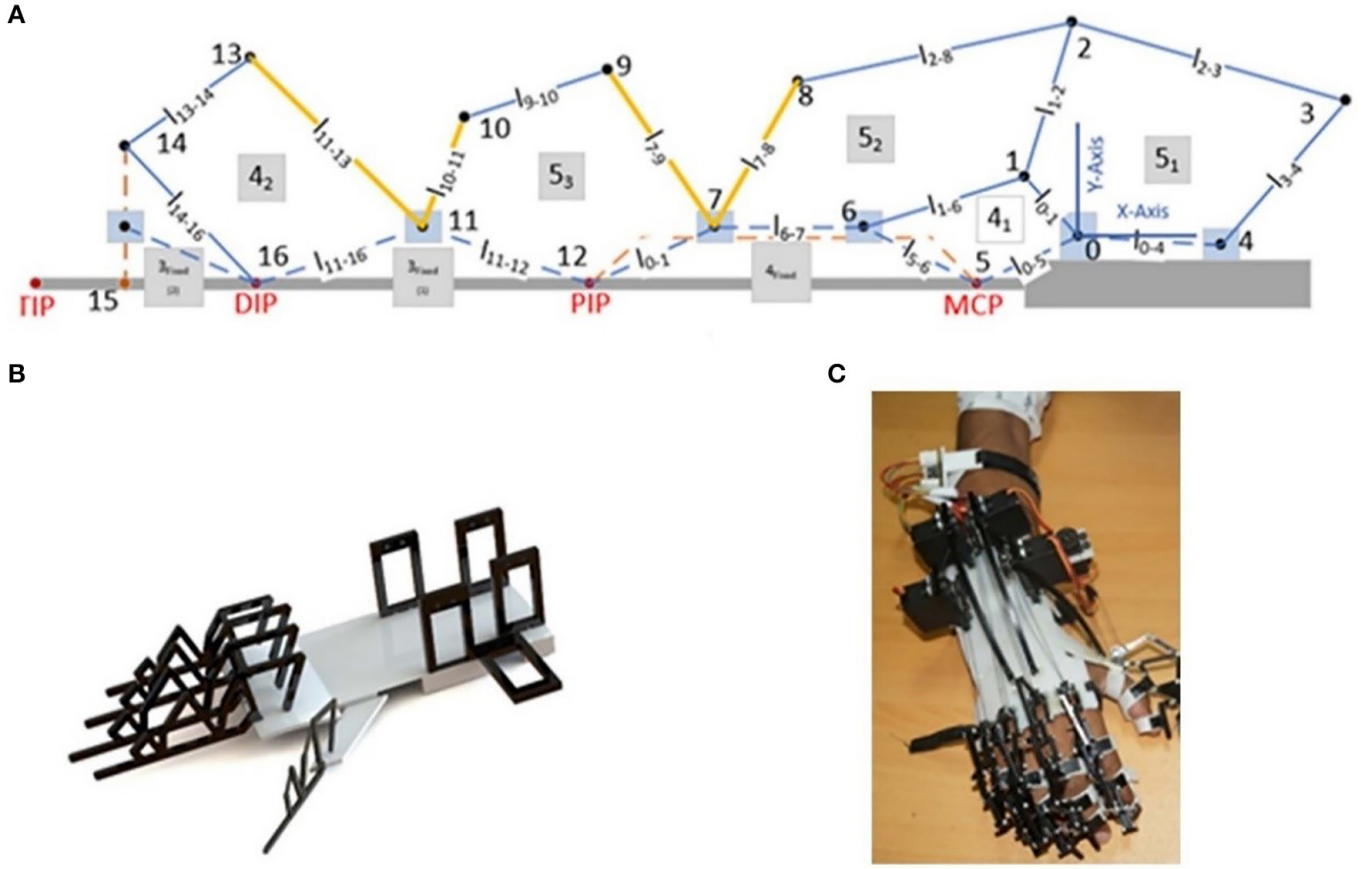
**(A)** Kinematic model, **(B)** CAD model, and **(C)** fabricated model.

In this study, the RISE Lab-SMME locally built robotic exoskeleton hand is used. This exoskeleton hand novel design is completely wearable and capable of controlling five (5) fingers separately. Each finger's movement is controlled by one servo motor mounted on the hand's backside, as shown in Figure 3. An indigenously developed five-degree-of-freedom (DOF) exoskeleton hand used in this research is shown in Figure 3B. The device features a servo-tendon actuation for controlling each finger's position and the thumb allowing various grasping motions and poses complying with those needed for daily life activities (ADL). The proposed mechanism has a five-DOF design with each of the finger and the thumb being independently controlled by servo motors. The transmission mechanism comprises a combination of linkages and threads, utilizing the natural finger's joints. It has the following salient features:

– Top-mounted
– Axis alignment
– Effective force transfer
– Smooth trajectories
– Lightweight design

The mechanism used for the fingers and the thumb is shown in Figure 3A, where **l** i-j is the link length between joints i and j. Joint motion, by varying the angle at joint 4 (between linkages **l** 0–4 and **l** 3–4, called the actuating joint) of the mechanism, can be performed to move the individual finger from its normally open position to the full close position.

**Figure 4:**
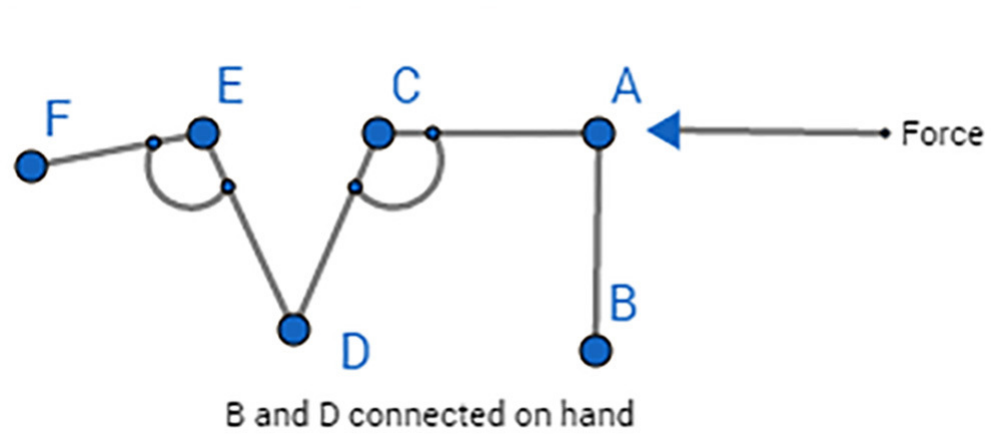
Conceptual depiction with V's and joint angles.

The joint angles between linkages **l** 7–8 to **l** 7–9 and **l** 11–13 to **l** 10–11 are both fixed. This allows the extra motion of the fingers as the hand closes. The main aim was to achieve better force and torque transmissibility at the end effector. Therefore, after manipulating different designs, we came up with the final mechanism design employing rigid V links for torque transmission, as shown in Figure 4. The force at the end effector changes as the fingers tend to move into a grasping position. Therefore, we tried to achieve an optimal design in which force has been optimized for the grasping position.

In Figure 4, when the rigid V link EDC rotates anticlockwise, i.e., the finger moves toward grasping position, angles FED and ACD tend to reduce toward 90°. This results in the maximum transmission of force from link AC to link EF because torque remains constant. Moreover, the force can also be increased from its initial value if the length of link ED is less than the link CD's length. Based on the linkage design, the joint motion ranges of the metacarpals (MCP), the proximal phalanges (PIP), and the distal phalanges (DIP) are shown in Table 1.

**Table 1:** Range of motion.

**Joint**	**Functional range of motion**	**Proposed design range of motion**
MCP	62.70	63.10
PIP	78.30	118.20
DIP	610	63.10

**Figure 5:**
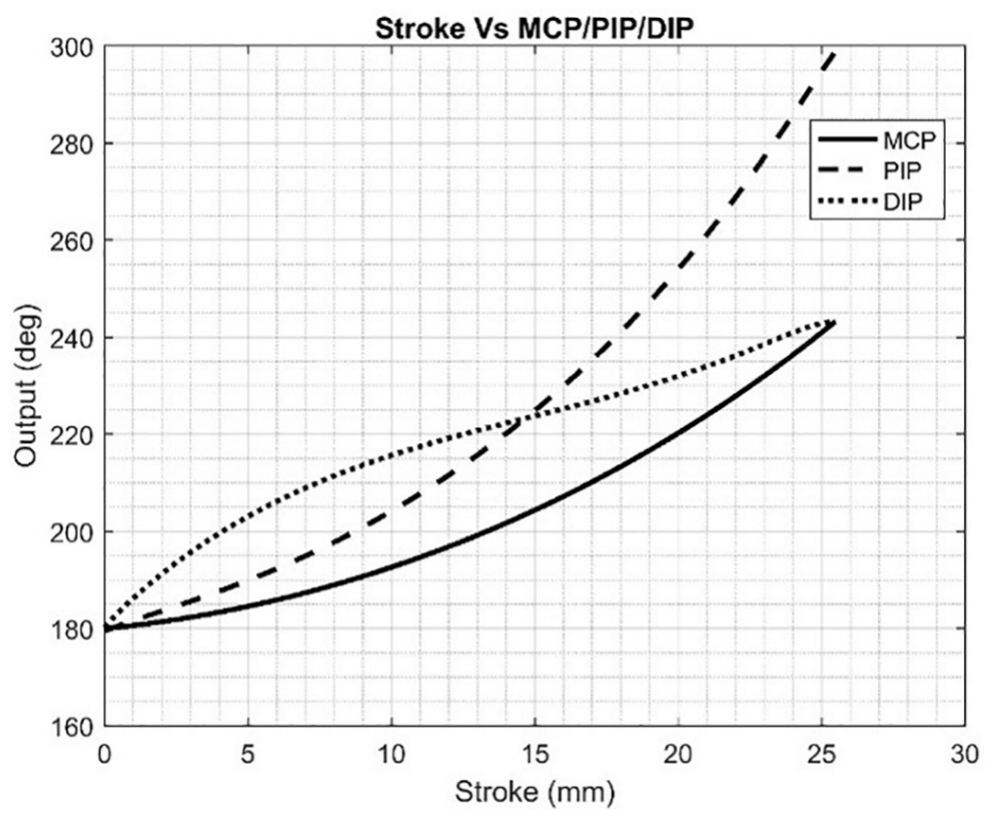
Stroke vs. joint angles of the exoskeleton hand.

**Figure 6:**
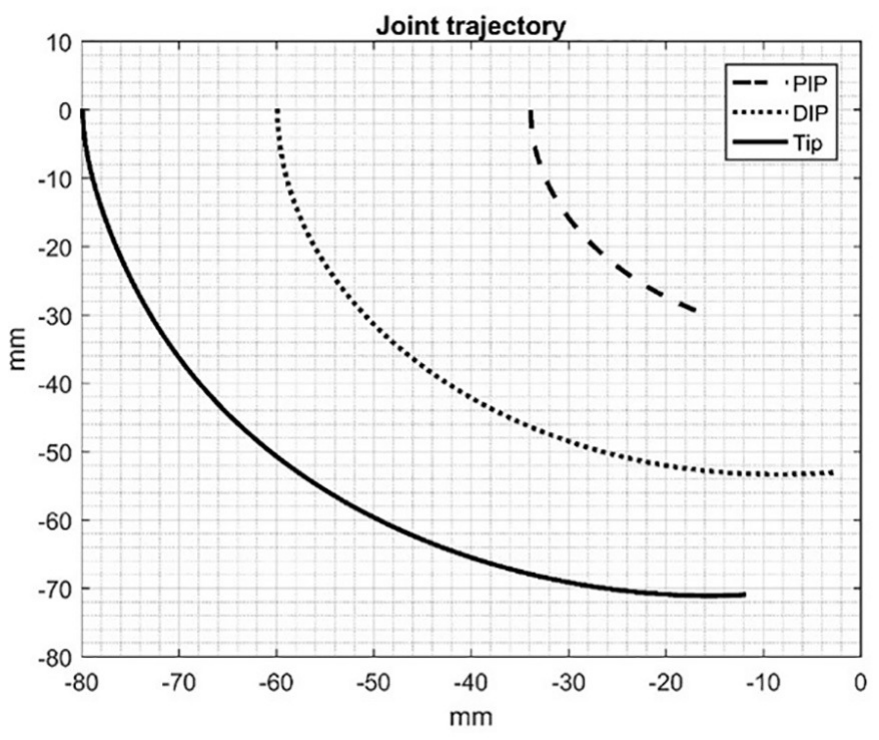
Joint trajectory of the exoskeleton hand.

The kinematics of the fingers based on the ranges of motion mentioned in Table 1 can be determined. Figure 5 shows the relation between actuator stroke in mm and MCP, PIP, and DIP joint angles for flexion or extension motion of the index finger. The extreme limits for the MCP, PIP, and DIP joint angles are 243.10, 298.20, and 243.10, respectively, for stroke equal to 25.5 mm. Figure 6 depicts the joint trajectories (PIP and DIP) and the trajectory of the fingertip. It also shows the smooth and logarithmic curve of the fingers.

**Figure 7:**
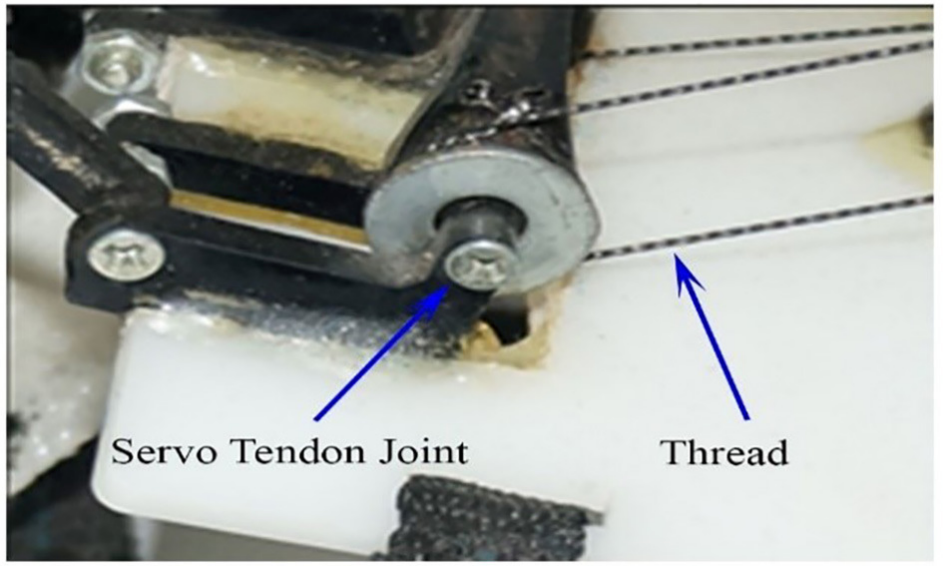
Double string arrangement.

The mechanism utilizes a unique servo-tendon actuation technique to drive the actuating joint. Tower Pro MG 995 servo motor was used because it produces enough torque, approximately 13 kg-cm. The actuators are subjected to stress during the system's functioning and need a suitable rigid base that holds them in place. The actuators were accommodated on the rear end of the hand's base. To completely lock the motors in place, slots of each motor dimension were cut, and then the motor was fixed in those slots with the help of a nut and bolt, providing a solid foundation for the actuators. The power transmission was a crucial decision in the execution of the project. As the system's efficiency was prioritized during the execution, the transmission decision was also primarily influenced by this scenario. The transmission's proposed options were as follows: A double string arrangement rotates the link **l** 3–4. In this arrangement, a strand of string was revolved around the servo pulley and then revolved around another pulley on the initial link. As the strings work under tension, they would not help return action to the initial form. Another strand was passed over the pulley and revolved in such a manner that it was opposite to that of the first string (Figure 7). This enabled tension above and below the pulley, and hence, a constant input of force was expected. The proposed system design is a soft exoskeleton-based BMI system having an easy plug-and-play interface. The system can be interfaced with different methodologies like EEG, fNIRS, and EMG. A video demonstration of the exoskeleton system in the Supplementary Video 1 exoskeleton hand application, is demonstrated while doing exoskeleton physical grasping tasks (glass lifting) using EMG signals showing different joint movements (DOFs) as a generalized assistive prosthetics technology.

### Brain–Machine Interface and Data Acquisition

**Figure 8:**
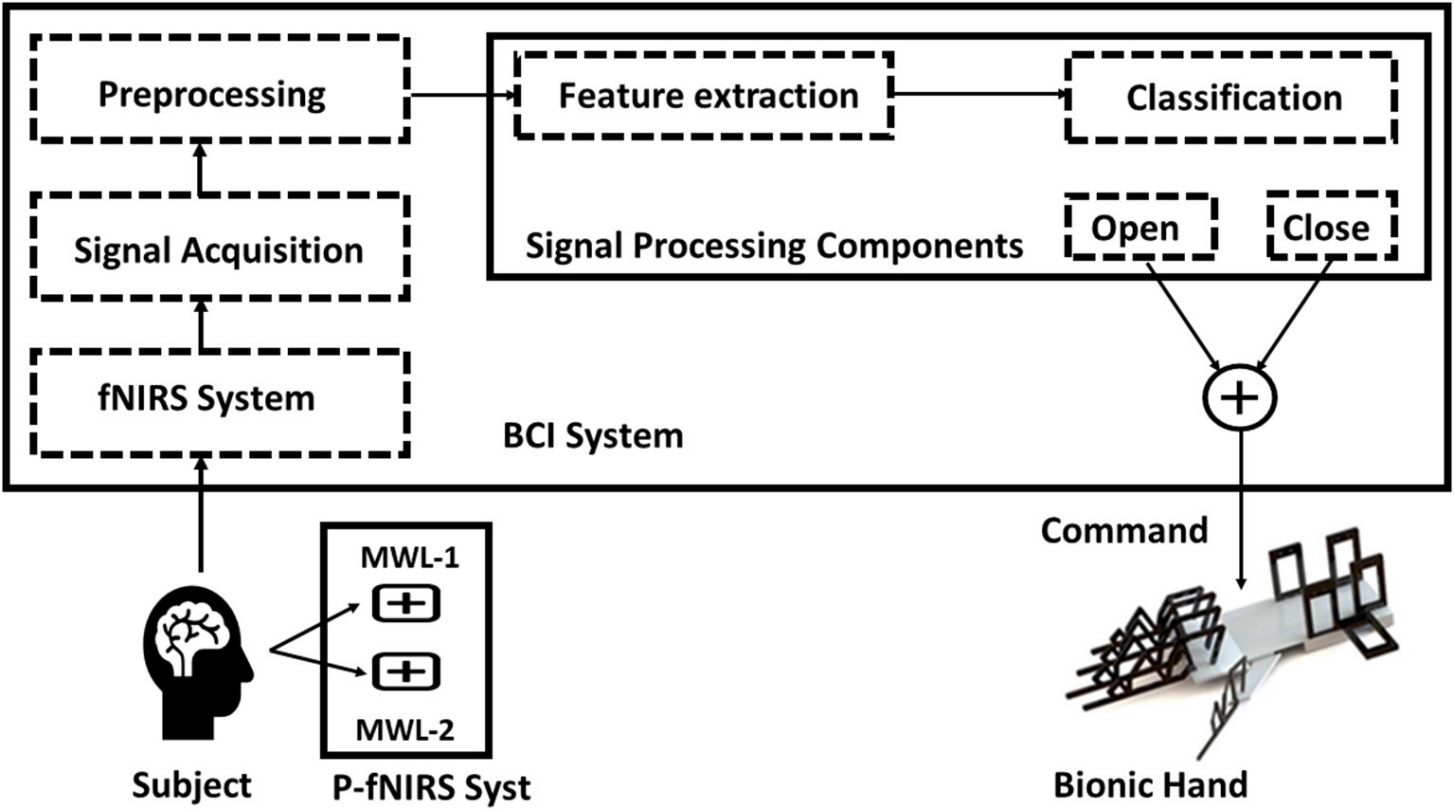
The BMI system architecture of the proposed method.

fNIRS headset P-fNIRSSyst (Asgher et al., 2019) is used, which the potential patient has to apply on the PFC area, for the data acquisition. The P-fNIRSSyst is a continuous-wave fNIRS system consisting of 12 channels arranged in an array-like structure, integrated with three near-infrared (NIR) sources having a dual-wavelength of 760 and 850 nm and eight photodetectors. The sampling rate of P-fNIRSSyst is 8 Hz. The fNIRS system estimates the brain's neuronal activity by measuring hemodynamic concentration changes in the PFC in the form of oxygenated (HbO) and deoxygenated hemoglobin (HbR). The features acquired brain hemodynamic concentration changes (ΔHbO and ΔHbR), which are then used to generate the BCI systems' commands. The complete architecture and system structural design is shown in Figure 8. The subject with the robotic exoskeleton hand wears the fNIRS device on the PFC region, which continuously measures hemodynamic concentration changes in the PFC, as shown in Figure 8. The proposed BMI system has several neuroergonomic features like an adaptable head band with PFC mounted curve design, lightweight, portable, easy integration with soft exoskeleton hand, independent finger movement, and comfort in a physical grasping action.

#### Experimental Paradigm

**Figure 9:**
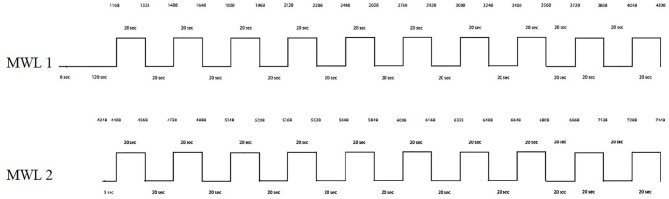
Experimental paradigm.

Participants were briefed about the tasks and wore both fNIRS PFC band and bionic arm connected with fNIRS signals. The subjects had to concentrate on mental math for a few seconds to induce brain activity, and then fNIRS signals were taken from the fNIRS headset and processed as mentioned in the benchmark study (Asgher et al., 2019). The experiments are considered to discriminate between difficulty levels of MWL. The mental arithmetic tasks are selected to evoke brain activities and employ a certain amount of MWL (Schudlo et al., 2013; Kosti et al., 2018). Participants restrict their physical movements to prevent artifacts and noise. Participants were presented with mental math task shown on the laptop screen placed at 70 cm. The initial 146 s were provided as a rest period to set the baseline (Asgher et al., 2020b). The baseline period is followed by MWL level-1, in which subjects performed mental arithmetic tasks for 20 s and then had 20 s of rest period. The same procedure was repeated for 10 trials. The MWL level-1 consists of a simple math task with three-digit addition with another three-digit number. MWL level-1 was modeled so that it provokes a minimal amount of MWL (Galy et al., 2012). After 10 trials of MWL level-1, subjects were presented with MWL level-2 with a delay of 25 s (base line). The MWL level-2 also follows the same pattern of 20-s activity and 20-s rest with 10 trials. The MWL level-2 contains arithmetic operations on equations and their answers (ans) being utilized in the next calculation (e.g., 768–5, ans ×4, ans −32, ans +912). MWL level-2 involves mental arithmetic tasks, short-term memory, and mental math (Herff et al., 2013; Hosseini et al., 2018). The difficulty level of MWL level-2 is greater than that of MWL level-1 and induces more MWL. For the confirmation of experimental paradigm, task difficulty of MWL tasks were gauged with the NASA-TLX. NASA-TLX is a subjective mental workload evaluation method to measure the cognitive loads in different environments. It is a multi-dimensional evaluation tool that rates recognized mental workload to assess the task difficulty and gauge its cognitive workload, effectiveness, and performance (Noyes and Bruneau, 2007; Paulhus and Vazire, 2007; Cao et al., 2009; Felton et al., 2012). The experiment followed a within-participants design with control variable conditions like participants' age limit and education level, and the order of the scenarios was counterbalanced using 10 consecutive trials in average, and the experimental paradigm is repeated, and the questionnaires were filled with subjects' input after MWL-1 and MWL-2, respectively. During the trials of MWL, the performance of participants remains at a satisfactory level (with less deviation). On the other hand, in case of fatigue or overload triggered by previous perceived stress, this may lead to impairment of performance (Kocalevent et al., 2011; Fan and Smith, 2017), which was not experienced in our case (trials). The performance of the participants was gauged both in time and accuracy. Moreover, the proposed methodology of fNIRS distinguishes different cognitive states and provides a robust gauge of mental effort measured from PFC and an effective method of measuring cognitive states (Peck et al., 2013). After the completion of the first task (MWL-1), participants filled out the NASA-TLX questionnaire and similarly for MWL-2. Results show the reliability of experimental tasks and the difficulty levels of two MWLs. The TLX (index) weight of MWL-2 >MWL-1 is also shown in the Supplementary Material (Results NASA Tlx MWL-1, and MWL-2). These outcomes are in line with the literature, in particular, an increased level of MWL is correlated with the tasks of very high difficulty (Rubio et al., 2004; Mansikka et al., 2019; Lowndes et al., 2020). The task timeline sequence of two MWL difficulty levels and rest period is shown in Figure 9.

#### Translation Algorithm and System Architecture

These processed signals are translated into two commands, i.e., “open” and “close” and then fed into the robotic exoskeleton hand, as shown in Figure 9. The experimental settings are designed to differentiate two levels of MWL. In previous studies (Schudlo and Chau, 2014; Kosti et al., 2018), mental arithmetic and programming tasks were used to provoke the brain and create a certain amount of MWL and can be used to generate discriminative signal feed to BMI systems. Targeted channels in this study are PF1, PF2, and PFz of the PFC region (Asgher et al., 2019). The designed pseudocode of the translation algorithm may consist of three main steps, along with an initialization state. In initialization, first, a vector variable is initialized along with three other pre-processor directives named “close,” “open,” and “tied.” In step 1, the data, in the form of MWL vectors read by serial read function. In step 2, previously read data is further checked using the “if” statement. Three “if” statements are used to cover “open,” “close,” and “tied” conditions. These two steps are repeated until the termination of the whole process (step 3). The complete algorithm is designed in MATLAB 2019a (MathWorks, Inc.).

#### Data Preprocessing for fNIRS Signals

Brain activity is detected by measuring changes in the concentration of oxygenated and deoxygenated hemoglobin (ΔHbO and ΔHbR). The modified Beer–Lambert Law (MBLL) was used for measuring concentration changes by using the intensities of detected NIR light at two different time instants (Pucci et al., 2010). The MBLL notation with change in concentration of HbO and HbR is shown in Equation (1).

(1) $$\begin{array}{r} \left[\begin{array}{c} \Delta C_{HbO}(t_{i})\\ \Delta C_{HbR}(t_{i}) \end{array}\right]=\frac{{\left[\begin{array}{cc} \alpha_{HbO}(\lambda_{1}) & \alpha_{HbR}(\lambda_{1})\\ \alpha_{HbO}(\lambda_{2}) & \alpha_{HbR}(\lambda_{2}) \end{array}\right]}^{-1}\left[\begin{array}{c} \Delta OD(t_{i};\lambda_{2})\\ \Delta OD(t_{i};\lambda_{2}) \end{array}\right]}{l\;x\;d} \end{array}$$

High-frequency artifacts (due to breathing, blood pressure, and heartbeat) are removed by passing signals through a low-passed fourth-order, zero-phased Butterworth filter (Franceschini et al., 2006; Barker et al., 2016). Brain hemodynamic fNIRS signals are passed through a low-passed band, fourth-order filter with a cutoff frequency of 0.3 Hz to remove high-frequency artifacts.

#### Hemodynamic Response Function

The temporal resolution of fNIRS usually depends on the properties of the underlying evoked neuronal and vascular changes. The time series blood oxygenated level-dependent (BOLD) response function depends on the nature of applied stimuli and hemodynamic response to neuronal events and is known as the hemodynamic response function (HRF). The metabolic rate in brain tissues increases with the activity and, as a result, increases the ΔHbO with a relative decrease in ΔHbR. The standard HRF shows the signal peaks during 5–8 s after triggering neuronal events, since neuronal activity increases metabolic demands that lead to an increase in the influx of oxygenated blood. Since the inflow of oxygenated blood continues and results in more supply than demand, the HRF becomes straightened roughly after 10–12 s (Naseer and Hong, 2015; Khan and Hong, 2017). The HRF in this study was calculated by spatially averaging across all channels and then temporally averaging the obtained vector from the previous step with respect to the number of trials, i.e., 10 for each MWL state.

#### Statistical Significance and Data Modeling

**Figure 10:**
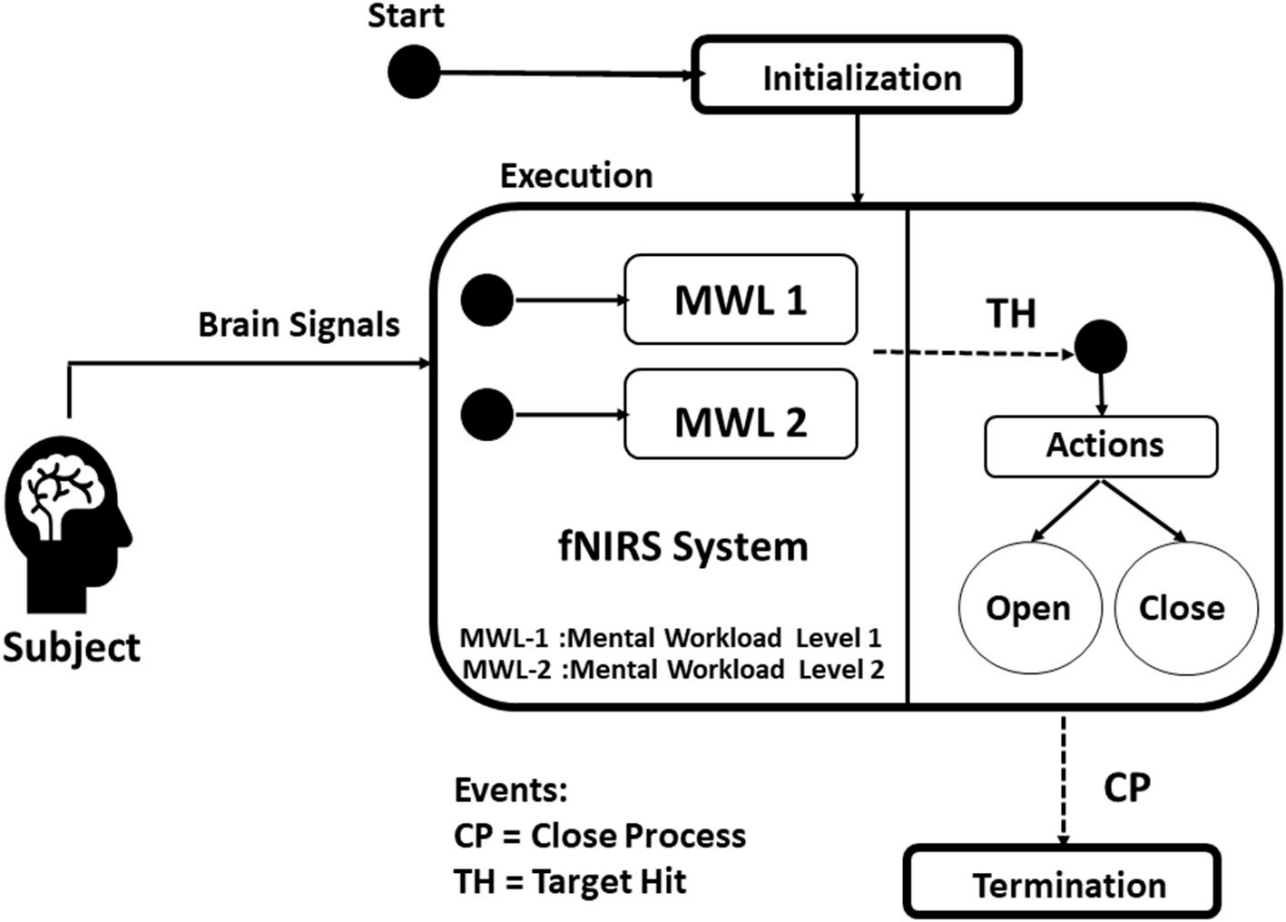
Experimental setup with bionic control.

The acquired hemodynamic signals can be affected by external and internal factors from accusation through optodes, transmission from fNIRS device to the computer, artifacts (Meyers waves, breathing), and the channel noise. To determine the validness of data acquired from the P-fNIRSSyst system, and that each channel has significant information, a statistical significance of data per channel is computed to measure the bad channel rejection. Independent-sample *t-*test and *p*-test were performed on the channel data. A discrete signal having an equal step size of mental workload activities (20 s) and the rest period is modeled and further compared with the acquired data to gauge the statistical significance. Data from only those channels are considered, which fulfill the criteria (*p* < 0.05). The percentage threshold under which the alternate hypothesis is deemed valid, known as alpha, is set to 5%. The data significance per channel computed from the acquired data is set at a threshold of 89.16% for a channel to be significant, as mentioned in detail (Asgher et al., 2020b). The channel numbers 1 and 10 did not satisfy this criterion and were excluded from further analysis. After data cleaning and bad channel rejection, and data modeling, the final data of 14 subjects are used in the final analysis, as shown in Table 2 and Figure 10. The obtained signals were also visually inspected for any artifacts like muscle movement effects, optode slippage, and other motion artifacts. The data recording of a subject with more than 10% contamination was excluded from further analysis. The data are analyzed and modeled based on the standard fNIRS data. The changes in concentration of ΔHbO and ΔHbR are plotted to depict the hemodynamic response function (HRF) by taking the spatial average of HbO and HbO at channels. The channels at which the HRF with the MWL is not exactly recorded due to the channel noise, and a second standard mathematical model of HRF (having ΔHbO rise in the activity region) of the signal is used to construct the HRF of the acquired data and applied on the data per channel. This allows the signal data during brain activity to be modeled close to the actual standard HRF model and discriminated for further utilization in the translation commands signals for the BMI system.

**Table 2:** Evaluated parameters (accuracy and ITR) of the proposed system.

**Subjects**	**Gender**	**Accuracy HbO**	**Accuracy HbR**	**Accuracy HbT**	**ITR HbO**	**ITR HbR**
S1	Male	89.05	87.53	88.29	1.45	1.43
S2	Male	89.11	86.83	87.97	1.44	1.40
S3	Female	91.31	88.84	90.07	1.52	1.47
S4	Male	91.47	88.87	90.17	1.52	1.47
S5	Male	86.90	87.97	87.43	1.42	1.44
S6	Female	85.66	84.35	85.01	1.34	1.31
S7	Male	89.63	87.74	88.68	1.47	1.43
S8	Female	88.69	86.89	87.79	1.43	1.40
S9	Male	89.01	88.98	88.99	1.48	1.48
S10	Male	80.15	83.75	81.95	1.24	1.29
S11	Male	90.09	89.47	89.78	1.51	1.50
S12	Female	83.99	86.66	85.32	1.35	1.39
S13	Female	89.90	88.99	89.44	1.50	1.48
S14	Male	85.67	86.51	86.09	1.37	1.39
Average		87.9 ± 3.01	87.38 ± 1.65	87.64 ± 2.24	1.43	1.42

## Feature Engineering and Classification

The standard procedure is to extract features from fNIRS data, and the extraction is directly carried from concentration changes of ΔHbO and ΔHbR. Features are selected based on data information in terms of important details; these features contain subsequent optimal classification (Naseer et al., 2016). Different types of features can be extracted from acquired hemodynamic signals. These features are calculated from temporal ΔHbO and ΔHbR data. The most commonly used features having maximum data information are signal mean, signal slope, signal variance, signal skewness, kurtosis, and signal peak reported in various studies (Naseer and Hong, 2015; Naseer et al., 2016) were also computed in this study.

### Mean

The arithmetic mean is a single value referring to the central tendency of given data. It is determined by the sum of all the data points over the total number of data points. Mathematically, the mean is expressed as:

(2) $$\begin{array}{r} \mu =\;\frac{1}{n}\;\sum_{i=1}^{n}x_{i} \end{array}$$

where x refers to the data points of a given data set from 1 to n.

### Standard Deviation

Standard deviation is used to quantify the dispersion of data points within a distribution from a mean value. Statistically, it is calculated as:

(3) $$\begin{array}{r} \sigma =\;\sqrt{\frac{1}{n-1}\;\sum_{i=1}^{n}{\left(x_{i}-\;\mu \right)}^{2}} \end{array}$$

where x refers to the data points from 1 to n; n refers to the total number of data points, and μ is the arithmetic mean of the data set.

### Variance

Variance is the squared standard deviation used to quantify the deviation of data points of a distribution from the mean value. Mathematically, the variance is expressed as:

(4) $$\begin{array}{r} \sigma^{2}=\;\frac{1}{n-1}\;\sum_{i=1}^{n}{\left(x_{i}-\;\mu \right)}^{2} \end{array}$$

### Slope

The slope or gradient of a line is a value referring to both the steepness and the direction of the line. It is the ratio of the vertical difference to the horizontal difference between two distinct points on a line and thus mathematically expressed as:

(5) $$\begin{array}{r} m=\;\frac{\Delta y}{\Delta x} \end{array}$$

where, Δy and Δx are the vertical difference and horizontal difference between two points on a line, respectively.

### Kurtosis

Kurtosis is the measure of peak around the mean distribution. Mathematically, it is calculated using the equation:

(6) $$\begin{array}{r} Kurtosis=\frac{\sum_{i=1}^{n}\frac{{\left(x_{i}-\;\mu \right)}^{4}}{n}}{\sigma^{4}} \end{array}$$

where x_i_ is the data points of the distribution, μ is the mean, σ is the standard deviation of the distribution, and n is the sample size.

### Skewness

Skewness is the measure of the asymmetry of a distribution about its mean. It can be positive, negative, or undefined. Mathematically, it is calculated using the equation:

(7) $$\begin{array}{r} Skewness=\frac{\sum_{i=1}^{n}\frac{{\left(x_{i}-\;\mu \right)}^{3}}{n}}{\sigma^{3}} \end{array}$$

where x_i_ is the data points of the distribution, μ is the mean, σ is the standard deviation of the distribution, and n is the sample size.

### Feature Extraction and Support Vector Machines

There are several possible features, features of which ones with the maximum data information are utilized in this study. In this study, commonly used features from HbO and HbR are calculated. After computing several feature combinations (signal mean, signal slope, signal variance, signal skewness, kurtosis, and signal peak) and the corresponding SVM accuracies, only two features, mean and slope, are selected due to their optimal feature combination for classification eventually. These features were spatially computed across significant channels with a moving overlapping window of 2 s. With a set of labeled training data in supervised learning, SVM draws hyper-planes to separate the closest training points (support vectors) with maximum distance outputs assigned to the categories of classification (Fernandes de Mello et al., 2018). The SVM, a powerful machine learning classifier, is employed for classification and is given in Equation (8). It maximizes the distance between the closest training points with a separating hyper-plane.

(8) $$\begin{array}{r} f\left(x\right)=r.x+b \end{array}$$

b is the scaling factor and r, x ϵ. The loss function of SVM is given in Equation (9)

(9) $$\begin{array}{r} J(\theta )=\sum_{i=1}^{m}y^{\left(i\right)}Cost_{1}(\theta^{T}(x^{\left(i\right)})+(1-y^{\left(i\right)})Cost_{0}(\theta^{T}(x^{\left(i\right)}) \end{array}$$

In Equation (9), m represents the total number of data points. The cost function is given by:

(10) $$\begin{array}{r} Cost(h_{\theta }(x),y)={\{}_{\max(0,1+\theta^{T}x)\;if\;y\;=0}^{\max(0,1-\theta^{T}x)\;if\;y\;=1} \end{array}$$

The data are classified with SVM into two categories of MWL-1 and MWL-2 based on the features. The labeled MWL trails are compared with performed actions, and the classification accuracy is determined with ΔHbO and ΔHbR and total hemoglobin (HbT), as shown in Table 2. In the study, the models of classification were subject specific. For the train-test split analysis, different paradigms including leave-one-out cross-validation (LOOCV), K-fold cross-validation, and 70:30 train-test split ratio were considered and analyzed. The purpose of the train-test split is to avoid overfitting of the model and checks how well a model generalizes to new unseen data from the same distribution. Each of the train-test methods has their own advantages; the 70:30 train-test split ratio for subject-specific data was used in this study. This avoids the distributional mismatch of the subjects' data in training and testing datasets. The leave-one-out cross-validation and K-fold cross-validation were not used because they were computationally and time expensive (Fushiki, 2011; Xu and Goodacre, 2018; Vabalas et al., 2019; Farias et al., 2020). Complete classification algorithms were trained and tested on the system MSI GE62VR Apache Pro Laptop with NVIDIA GEFORCE® GTX 1060 having a 3GB GDDR5 graphic card. SVM classification was performed on MATLAB 2019a (MathWorks, Inc.) using the machine learning application.

#### Data Transfer Rate and Information Transfer Rate

The data transfer rate (DTR) and information transfer Rate (ITR) are the assessment metrices used in BMI studies to estimate the amount of information in bits passed on by the system's output to operate the interface and evaluate the applicability of classification (Obermaier et al., 2001; McFarland et al., 2003). ITR was first introduced in information theory and used to quantify the reliability of information (Obermaier et al., 2001) to gauge the number of mental tasks with the reliability of classification accuracy and the rate at which the information or command signals are translated to the robotic exoskeleton arm. DTR and ITR depend on the number of classes, task duration, and the classification accuracy. fNIRS-ITR can be increased with increasing number of classes, but with more classes, the classification accuracy decreases. Multi-task classification in this study with two classes (two-state MWL) with appropriate accuracy and task duration are employed to generate considerable ITR to operate the BMI system. In case of data transfer, EEG is preferred over fNIRS, and the ITR of EEG and EMG are large compared with the fNIRS owing to its less sampling frequency and temporal resolution (Obermaier et al., 2001; Power et al., 2012; Shin and Jeong, 2014). DTR is denoted by Bm and is calculated using ITR (Bt—bits/trail):

(11) $$\begin{array}{r} \text{Bt }=\;\log_{2}\text{N }+\;\text{P }\log_{2}\;\left(\text{P}\right)+\;\left(1-\text{P}\right)\log_{2}\frac{1-\text{P}}{\text{N}-1} \end{array}$$

where N is the number of targets (stimulus) or number of classes, and P is the classification accuracy. DTR is computed in bits per minute using Equation (12), where Cn is the number of classifications, and T is the processing time in second (Wolpaw et al., 1998; Erkan and Akbaba, 2018).

(12) $$\begin{array}{r} Bm=\frac{60}{T}Cn.Bt \end{array}$$

## Results

Each subject undertook 10 trials for each MWL difficulty level. The subject had to evoke mild mental activity (open action) first and then higher and intense mental activity (immediate action to close action).

**Figure 11:**
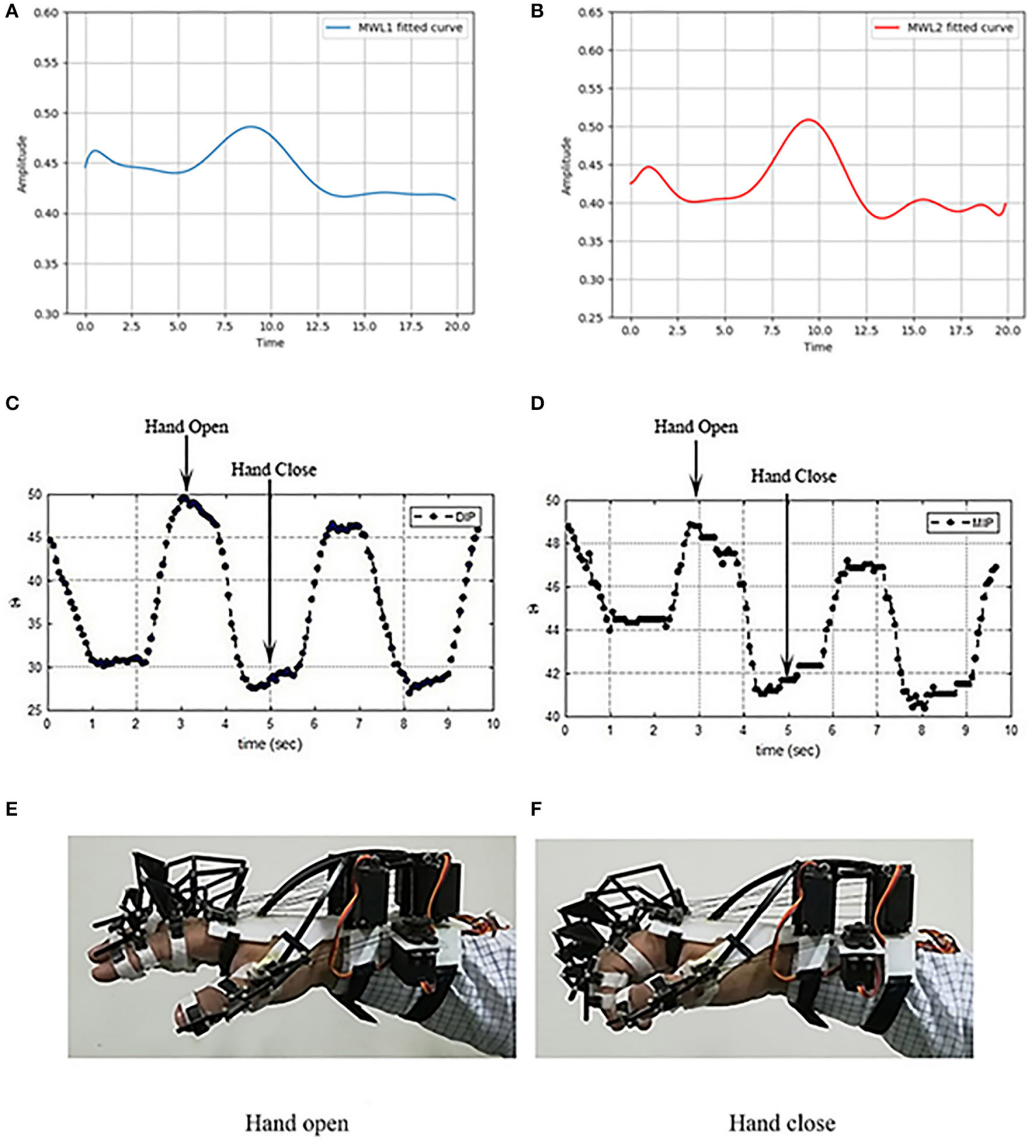
**(A)** The hemodynamic response function (HRF)–fNIRS signal at mental workload (MWL)-1 (hand open), **(B)** the HRF-fNIRS signal at MWL-2 (hand close), **(C)** distal phalanges (DIP) joint's plot against the opening command, **(D)** MIP joint's plot against the closing command, **(E)** exoskeleton hand opened, and **(F)** exoskeleton hand closed.

The SVM classifier is used on channels having considerable statistical significance and calculated in the previous step (*Statistical Significance and Data Modeling* section). The total length of the recorded fNIRS signal is 546 s. The averaged accuracy achieved through HbO, HbR, and HbT is 87.9% ± 3.01, 87.38% ± 1.65, and 87.64% ± 2.24, respectively. ITR is calculated from HbO, HbR, and HbT to examine the successful information transferred for the deigned soft exoskeleton system. The results attained with HbO and HbR are shown in Table 2. The averaged ITR achieved with HbO is 1.43. The MWL control signals are applied to the exoskeletal hand online, but the results show that real-time testing can be applied but with a limited capability of ITR compared with EEG, which has high ITR and faster control translations (Spüler, 2017; Xing et al., 2018). The analysis is not real time; however, this limitation is due to the inherent limitation in fNIRS with small ITR that takes some time to process the data. The HRF plots of acquired fNIRS signals are shown in Figures 11A,B, controlling commands for open and close after pre-processing, and Figures 11C,D show the corresponding opening and closing angles of hand's MIP and DIP joints data and implementation on exoskeleton hand as mentioned in Figures 11E,F.

## Discussion

**Figure 12:**
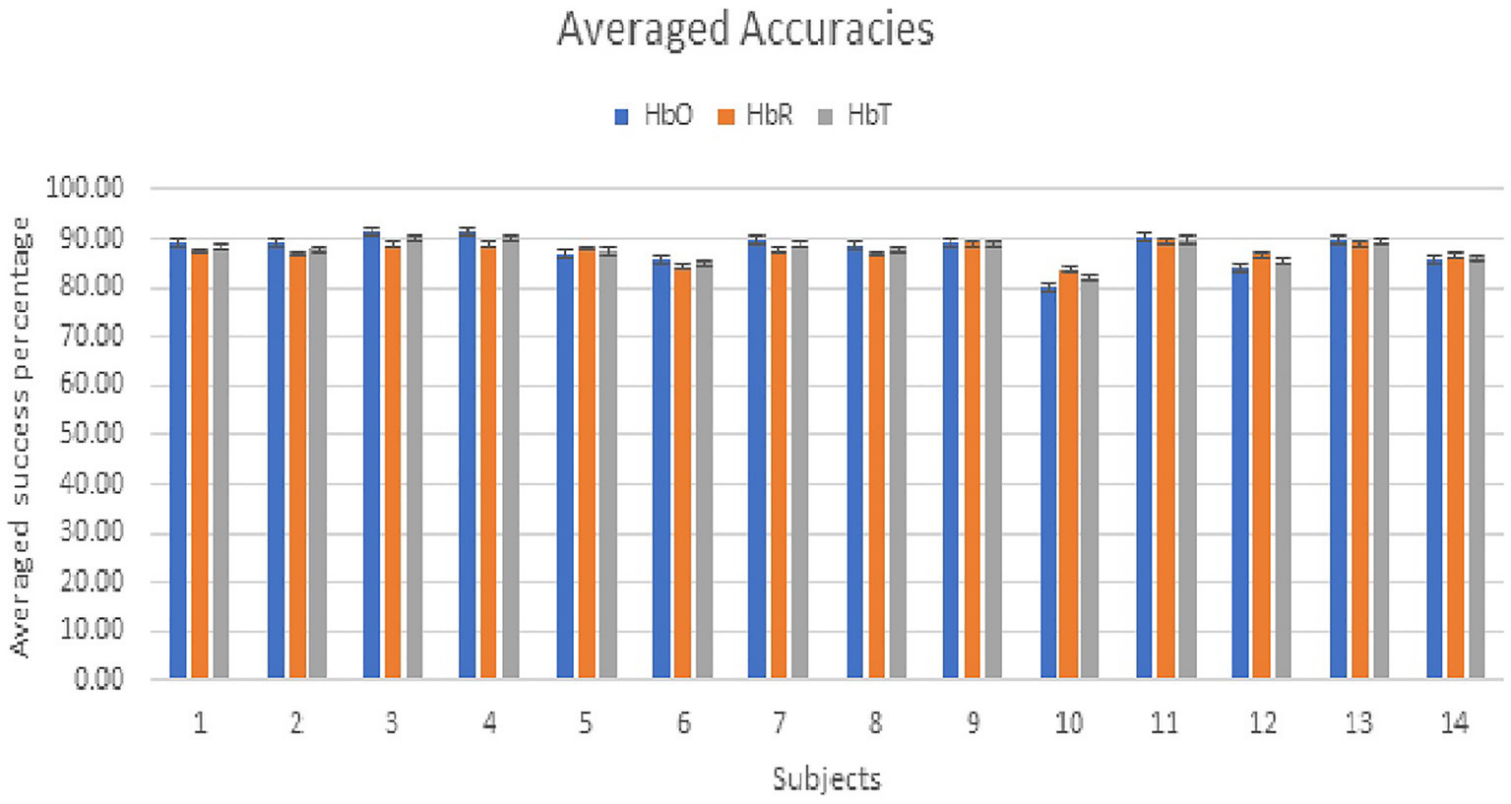
The average performance and accuracies of all subjects against (open–close) exoskeleton hand commands.

In this study, a wearable soft exoskeleton hand is designed, and its controlling technique proposed for the potential patients suffering from a stroke or severe disability for grasping tasks. A low-cost servo tendon-driven exoskeleton hand is designed, which is controlled using the proposed fNIRS-based MWL paradigm, and each finger can move independently, unlike the previous studies (Shahid et al., 2018), where all fingers were tied except the thumb in most of the studies (Ramadan and Vasilakos, 2017; Wang et al., 2019). In this study, conversion of low and high MWL was utilized as operational commands in opening and closing of the robotic exoskeleton. MWL is subjective, and signals from PFC are cognitive in nature. MWL is considered as a specific task that could be utilized in the BMI especially in situations like overload, fatigue, and stress, and to evoke a functional brain response. Sixteen right-handed (11 males and 5 females) healthy participants took part in this research. After screening and bad channel correction, the accuracy performance and ITR of the final 14 subjects are shown in Table 2. Figure 12 shows that both operations were performed equally by each subject, with an average performance accuracy of 87.9%. The total length of the recorded fNIRS signal is 546 s. The maximum accuracy achieved is 91.31%, while the minimum accuracy is 80.15%, as shown in Table 2 and Figure 10. The major takeaway of the system is the brain–machine interface (BMI) aspect in the form of a portable PFC fNIRS-based interface with lightweight exoskeletal hand, and limited non-ergonomic advantages are the system's portability, MWL applicability, ease of use, curve shape fitting on PFC, and translating controls to the lightweight exoskeletal hand. Since the main neuroergonomic advantages to the exoskeleton are the human brain at work using MWL, we tried to design the soft exoskeleton hand that may assist a person's daily working, whether in physical grasping or holding tasks (von Lühmann et al., 2015; Lotte and Roy, 2018). In the case of a stroke patient, when he or she tries to generate operational commands, the neural blood flow and pathways of the patients are different in case of the injury or disease (Birbaumer and Cohen, 2007; Chodobski et al., 2011; Käthner et al., 2017). MWL is a proposed task that could be applied even under stress and cognitive load, and the patients could generate control command signals to operate the exoskeleton. The results obtained using NASA-TLX and fNIRS MWL data pattern validate the experimental paradigm for assessment and analysis. The overall focus of the study is the design for a soft exoskeleton system interfacing with the brain's PFC communication in an ecological environment.

Many researchers (Brose et al., 2010; Meng et al., 2016; Zhang et al., 2017; Chen et al., 2018) presented different techniques in similar research areas and used different neuroimaging techniques. The relevant research is presented in a few EEG and fNIRS studies (Li et al., 2017; Shin et al., 2017; Khan et al., 2018) for gait rehabilitation and increasing accuracy for the BMI system. In this study, a BMI system is proposed, which pertains to physical hand grasping tasks in a controlled environment. In Rea et al. (2014), used the fNIRS signal to detect lower-limb movement for gait rehabilitation. They acquired fNIRS signals in stroke patients during preparation for hip movement with 67.77 ± 11.35% accuracy. Zhao et al. (2017) proposed a prosthetic controller to control and operate a bipedal robot. A walking gait cluster pattern was initiated for the robotic system, and an online, optimized transfemoral prosthesis control technique [control Lyapunov function (CLF)-based quadratic programs (QPs)] was examined on the knee and ankle joints of the prosthetic device. Perrey (2014) extensively studied the neural gait control using fNIRS. The proposed system was not real-time tested, and the offline classification is performed on the healthy subjects in a lab. The MWL control signals are applied to the exoskeletal hand online. The results show that real-time testing can be applied but with a limited capability of ITR compared with EEG, which has high ITR and faster control transitions (Spüler, 2017; Xing et al., 2018).

Similarly, in Ortner et al. (2011), the authors used SSVEP for rehabilitation (the channel used was O1 along with Fz as a reference node at 256 Hz sampling rate), and the accuracy reported was more than 60%, while the proposed fNIRS-BMI technique's average accuracy is 87.9% at 1.43 ITR. The ITR can be further improved by reducing the task duration or number of classes, but in that case, the accuracy would reduce. The tradeoff between performance accuracy and ITR is tried to be placed near-optimal in the proposed system design. Li et al. (2017) proposed a hybrid EEG-fNIRS BCI system's highest classification accuracy of 91.02 ± 4.08%, while using the EEG classification accuracy of 85.64 ± 7.4%, and with fNIRS, the study stated an average accuracy of 85.55 ± 10.72%. The authors in Ortner et al. (2011) used PSD along with discrete Fourier transform (DFT). More detailed data of previous studies and comparisons are presented in Table 3, including classifier, hardware, electrodes, and nature of the study. Ortner et al. (2011) and Downey et al. (2016) had presented the wearable exoskeleton hand, but all fingers were tied, making the upper part of the hand fixed like a palm to reduce the number of actuators. In this proposed study, we tried to address this issue by eliminating this limitation, and all fingers of the exoskeleton hand are capable of moving independently as shown in the Supplementary Video 1 exoskeleton hand application.

**Table 3:** Comparison of brain–machine interface (BMI) and prosthetics control studies.

**References**	**Classifier**	**Hardware**	**Electrodes**	**Technique**	**Brain activation**	**Wearable/non-wearable**
Chen et al. (2018)	CCA	7 DOF Robotic arm	P3, Pz, P4, PO3, PO4, T5, T6,O1, Oz, and O2	SSVEP	Neural	Non-wearable
Zhang et al. (2017)	CNN	ID-SIR system	FPz, Oz	SSVEP and P300	Neural	-do-
Meng et al. (2016)	Event related synchronization/desynchronization	6 DOF Robotic arm	C3, C4	Thoughts/imagination	Neural	-do-
Brose et al. (2010)	^***^	Wheelchair/robotic manipulator	^***^	^***^	Neural	-do-
Downey et al. (2016)	BMI decoding	Robotic Manipulator	^***^	BMI and Comp. Vision	Muscles and Neural	-do-
Fukuma et al. (2018)	Variational Bayesian multimodal	Prosthetic hand	^***^	MEG/eSCP	Muscles and Neural	-do-
Yang et al. (2018)	SVM	Robotic Manipulator	Pz, P3, P4, PO3, PO4, PO7, PO8, Oz, O1,O2	SSVEP	Neural	-do-
Müller-Putz and Pfurtscheller (2008)	DTF/HSD Motor imaginary	Prosthetic hand	O1 and O2	SSVEP	Neural	-do-
Looned et al. (2014)	Linear/binary classifier	Upper extremities (UE)	All (14 channels)	Functional electrical stimulation	Neural	Wearable
Rea et al. (2014)	LDA	Lower limb movement	All (48 channels)	fNIRS	Hemodynamic	-do-
Ortner et al. (2011)	Weighted PSD along with discrete FF	Prosthetic hand	O1, and Fz	SSVEP	Neural	-do-
Khan et al. (2018)	LDA and SVM	Prosthetic leg	Left hemisphere of M1	fNIRS	Hemodynamic	-do-
Costa et al. (2016)	LDA, SVM, K-NN, NB, and DTL	Participant's attention to the gait	Significant channels	EEG with gamma band	Neural	-do-
Borgheai et al. (2020)	LDA with single-trial Visuo-Mental (VM)	Amyotrophic lateral sclerosis (ALS)	F1, F2, AFz, Fp1, and Fp2	fNIRS	Hemodynamic	-do-
Proposed method	SVM with mental math task	Five DOF with independent control Prosthetic hand	PF1, PF2, and PFz	fNIRS	Hemodynamic	-do-

*** *The specific details were not mentioned in those research studies*.

In BMI, the research studies are primarily focused on validating the experimental paradigm, authenticating the new custom-built devices, procedures for controlling devices, verifying the applicability of hardware for healthy participants or patients (stoke, motor disability, ALS), and conducting experimental lab trials on healthy patients and reporting their findings and results (Abiri et al., 2019). Shirley Coyle et al. designed a simplified fNIRS device, while Wyser et al. designed a wearable modular fNIRS device, and both studies were tested and findings validated on healthy subjects (Coyle et al., 2007; Wyser et al., 2017). In different studies, Noah et al., Oh et al., and Asgher et al. designed and validated their experimental paradigms: naturalistic task, attentive locomotion task, and mental arithmetic task, respectively, performed on healthy participants (Noah et al., 2015; Oh et al., 2018; Asgher et al., 2020b). In another study, the authors designed an fNIRS-based neurorobotic interface for gait rehabilitation and reported findings on healthy patients (Khan et al., 2018). Costa et al. (2016) evaluated an association among the cortical signals and the cognitive mechanisms associated with the attention during gait using offline analysis on healthy participants and SCI patients, and analyzed their brain activation. Similarly, Magosso et al. (2019) analyzed alpha rhythm and detected changes in attention during human interaction with an artificial environment, using EEG with a framework for the potential end users (patients). The reported accuracy in the case of patients is slightly less compared with the healthy patients owing to differences in neural and hemodynamic patterns either due to disease or injury. The proposed methodology is unique as it is designed, keeping in view both BMI and ergonomic factors. The first significant lead is a wearable and lightweight servo tendon-driven design of an exoskeleton hand with a portable fNIRS system for brain data (MWL) acquisition. Second, all fingers are operated separately along with the thumb, and they can move independently with one actuator for each finger. Third, the overall average accuracy is comparable and, in some cases, higher than previous similar studies (Müller-Putz and Pfurtscheller, 2008; Ortner et al., 2011; Looned et al., 2014). A comparative analysis is presented in Table 3. The direct comparison cannot be established between EEG and fNIRS, and the tasks are also not comparable because of several differences like spatial and temporal resolution and prominently the ITR in BCI and BMI applications. Several EEG studies show that a prosthetic hand can be controlled using brain signals, although the fNIRS-BMI studies on the soft exoskeleton control are very limited (Lalitharatne et al., 2013; Gao et al., 2016; He et al., 2018). In our study, by analyzing the TLX, it could be also possible to assess subscale scores affected by the mental workload related to a specific task. In experiments where mental demand is increased, participants mentioned higher perceived mental demand, effort, and frustration, with lower subjective perception of their task performance (Mingardi et al., 2020). The task duration of 20 s is required to acquire that data to generate the commands, and around 1 s is needed to execute that command. This may be a long duration for a healthy person, but for a stroke patient or amputee who cannot move the arms, this execution time may be useful for the patients to perform motor task on their own. Further experimentation with small task duration windows could help in faster command generation with time reduction while keeping accuracy and ITR at desired levels (Shin and Jeong, 2014). The comparison of the proposed system with existing EEG studies in Table 3 is mentioned to indicate that an fNIRS-based exoskeleton can be a potential research area for BCI and BMI applications. Borgheai et al. (2020) proposed an fNIRS-based BCI system that shows communication and control for late-stage ALS patients (lose voluntary muscle control) with a maximum average accuracy of 81.3 ± 5.7%.

The field of BMI is emerging as an assistive methodology and aiding patients with different disabilities (Min et al., 2010; Teo and Chew, 2014; Shik et al., 2020). Despite its potential role in neurorehabilitation, the practicality of BMI depends on the patient and environment specific to the patient, owing to the human brain's dynamic nature (Belda-Lois et al., 2011; Cervera et al., 2018). The need for recalibrating and adjusting the BMI system's settings for every new session with a new subject due to the patient's dynamic brain electrical and hemodynamic profile makes the actual applicability of BMI patient specific (Krauledat et al., 2007; Millán et al., 2010; Rieke et al., 2020). Accounting for the human brain's uncertain behavior is due to variations of in the users' mental state and psychological state, miss-concentration, attentiveness, and fatigue levels. It may also be influenced by numerous measurement conditions, such as the changes in the impedance of the electrodes due to sweating and other hardware or external environmental factors (Azab et al., 2018).

There are few limitations of the current study; the first is analysis is not done in real time, and instead, online classification is used on the participants' data. However, this limitation is due to the inherent limitation in fNIRS with small ITR that takes some time to process the data and generate command translation signals. The second limitation is that the study is conducted on healthy subjects and designed for potential stroke patients. Different studies have already demonstrated the feasibility of functional near-infrared spectroscopy (fNIRS) to successfully control BCIs primarily for healthy participants (Naseer and Hong, 2013, 2015; Borgheai et al., 2019). There are promising outcomes for BMI in healthy subjects. The evidences that BMI applications may also produce clinically significant motor recovery results after stroke and in people with motor disabilities are also reported in various studies. The accuracy reported in these studies for patients is slightly less compared with the healthy persons mainly due to the patients' specific experimental protocols compared with the healthy participants and the difference in the hemodynamic behavior of the brain (Burns et al., 2014; Costa et al., 2016; Käthner et al., 2017; Borgheai et al., 2020; Rieke et al., 2020). In ecological settings, the BCI systems face numerous challenges such as low BCI signal strength, low data transfer rate, and high error percentage due to high brain signal variance (Kameswara et al., 2012; Ramadan et al., 2015). The accuracy of BCI system is also effected and sometimes reduced owing to the lack of ability of the patient to retain similar cognitive states in various sessions as it is observed that long intervals of usage introduce cognitive fatigue for the patients (Papanastasiou et al., 2020). The tasks in this study were designed for potential stroke patients who may generate control commands even under stress and cognitive load. The signals are also affected by the person's eye blinks, muscular movements, and hearing sound (Cincotti et al., 2008; Abiri et al., 2019). In this context, a methodology is proposed in this study for stroke patients to utilize their cognitive load signals in the form of MWL, due to its strong association with the subject's performance and related stress (Aricò et al., 2016). Various fNIRS and EEG studies utilized mental arithmetic tasks and cognitive brain data from PFC in BCI and neurorehabilitation applications (Shih et al., 2012; Naseer and Hong, 2015; van Dokkum et al., 2015; Shin et al., 2016; Ayaz et al., 2018; Chaudhary et al., 2020). In similar studies, Khan et al. (2014) studied hybrid fNIRS-EEG for decoding four movement directions, the study stimulate the changes in concentration of HbO (acquired fNIRS signals) with MA task as forward and backward directional signals and changes induced in EEG through a hand tapping task as left and right directional signals. Further, Hong et al. (2018) examined the use of a hybrid (fNIRS-EEG) BCI for the patients with locked in syndrome.

In this study, first, we validate the BMI hardware (soft exoskeleton hand with the fNIRS system) design and experimental paradigm of a two-state MWL offline classification on healthy patients. The proposed system can be used as a benchmark study for future fNIRS-based BMI applications. The results suggest the plausible accuracy for fNIRS-based servo tendon-driven exoskeleton system. The exoskeleton has been used on healthy participants in the lab environment. However, the full system has to be tested in the future on actual patients with varying levels of MWL. Further experimentation with small task duration windows could help faster command generation with time reduction in future research while keeping the accuracy and ITR at desired levels. For future research, we would try to introduce the adaptive force control for grasping tasks and GUI for the physiotherapist's operational use. Also, we intend to extend our experiments and perform real-time classification on patients with motor disabilities. This study serves as the feasibility study for detecting two-state MWL with fNIRS and the execution of generated commands through a servo tendon-driven exoskeleton system.

## Conclusion

The integration of neuroergonomics with the brain–machine interface (BMI) systems is the need of the hour. This study is designed for potential stroke patients and performed online classification of mental workload (MWL) on healthy participants to gauge the BMI system's applicability. The brain data of participants are recorded in the form of MWL acquired using a custom-built fNIRS system and translated into a soft exoskeleton system, keeping the system's weight and portability as flexible as possible. Two MWL commands operate a lightweight bionic arm with a servo tendon-driven exoskeleton designed explicitly for hand-grasping tasks. Targeted channels and regional areas of interest on the pre-frontal cortex (PFC) are PF1, PF2, and PFz. The two-state MWL is recorded at 8 Hz sampling frequency and used to operate each finger (open and close) independently in the soft exoskeleton hand. Support vector machine (SVM) classifiers are used to generate command signals (open or close) for the prosthetic hand. The maximum classification accuracy is 91.31%, with an average accuracy of 87.9% and an average information transfer rate (ITR) of 1.43. The results show the effectiveness of the proposed brain–machine interface (BMI) system for potential patients having difficulties in grasping tasks.

## Data Availability Statement

The original contributions presented in the study are included in the article/Supplementary Material, further inquiries can be directed to the corresponding author/s.

## Ethics Statement

The studies involving human participants were reviewed and approved by Ethical Research Council of RISE lab at SMME-National University of Sciences and Technology (NUST). The patients/participants provided their written informed consent to participate in this study.

## Author Contributions

UA and MK conceptualized the study. UA, NN, and MK were in charge of the methodology. UA, KK, and MK handled the software and conducted the formal analysis. MK, MA, KK, and UA handled the validation. UA, MA, and MK conducted the investigation and wrote the original draft. RA, YA, and NN were in charge of the resources and handled the supervision. UA and KK revised and edited the draft. UA, NN, and YA handled the visualization. RA and YA were in charge of the project administration. All authors contributed to the article and approved the submitted version.

## Conflict of Interest

The authors declare that the research was conducted in the absence of any commercial or financial relationships that could be construed as a potential conflict of interest.
