# Supplementary material for: Motor Training Using Mental Workload (MWL) With an Assistive Soft Exoskeleton System: A Functional Near-Infrared Spectroscopy (fNIRS) Study for Brain–Machine Interface (BMI)
Source: Front Neurorobot. 2021 Mar 18;15:605751. doi: 10.3389/fnbot.2021.605751 (PMC8012849; doi:10.3389/fnbot.2021.605751)
Supplement: Supplementary file 3 [file Data_Sheet_2.PDF]

Raw data of Participant "ByAO1HXP8" in Experiment "2"

Experiment performed at April 2nd 2020, 3:35:08 pm

Weighted rating: 52

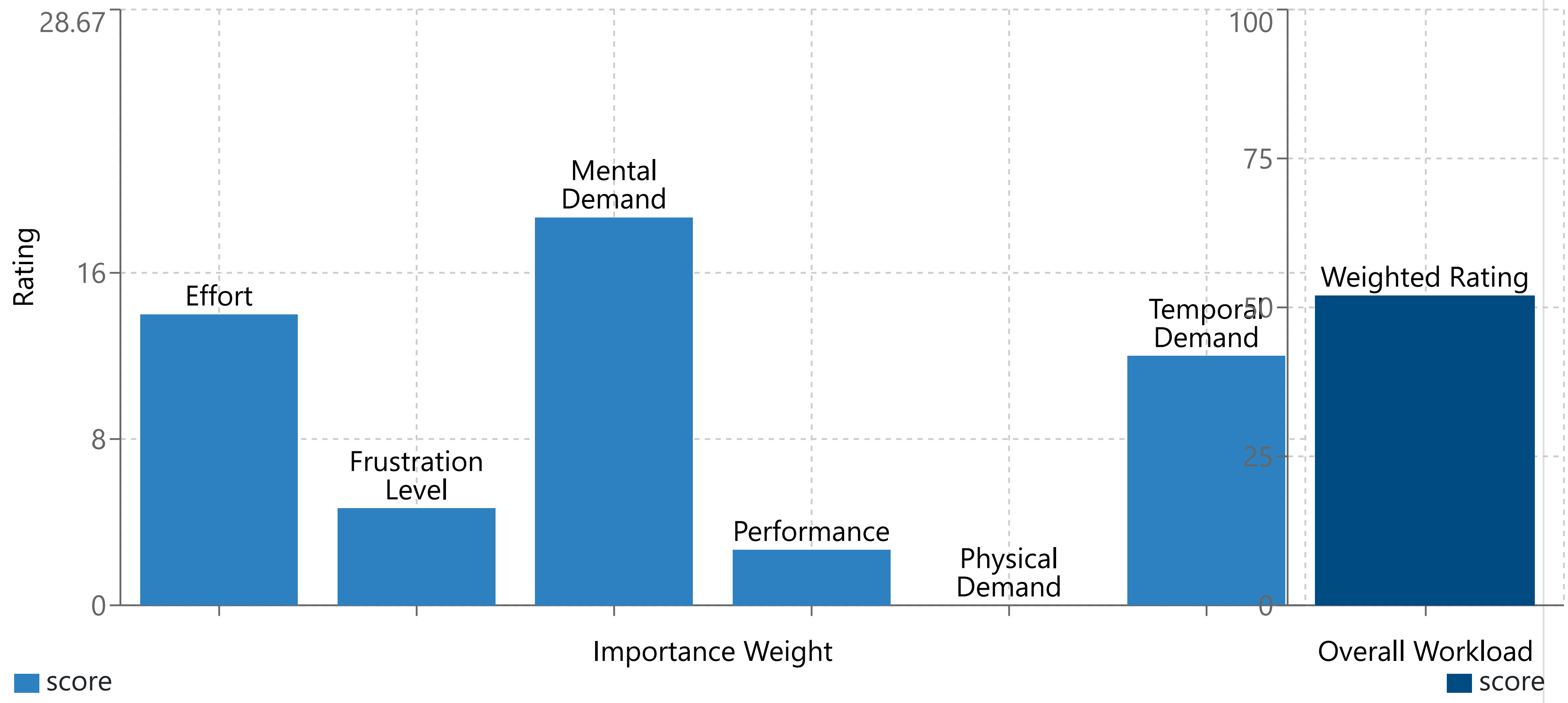

Raw ratings

Effort	Frustration Level	Mental Demand	Performance	Physical Demand	Temporal Demand
70	70	70	10	20	60

Sources of Workload tally (number of times selected)

Effort	Frustration Level	Mental Demand	Performance	Physical Demand	Temporal Demand
3	1	4	4	0	3

Adjusted Rating (Weight x Raw)

Effort	Frustration Level	Mental Demand	Performance	Physical Demand	Temporal Demand
210	70	280	40	0	180

Raw data of Participant "HJtNMB7DL" in Experiment "4"

Experiment performed at April 2nd 2020, 3:46:39 pm

Weighted rating: 80.67

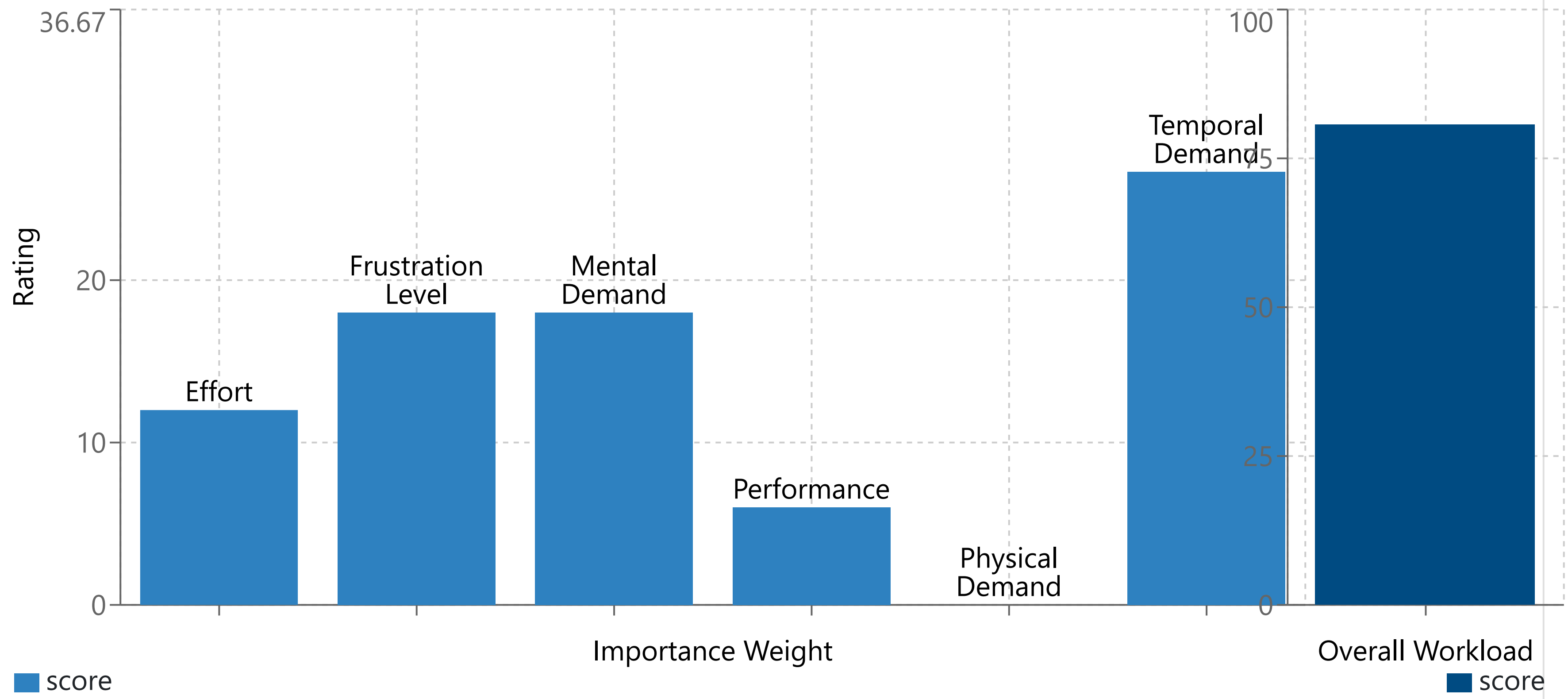

Raw ratings

Effort	Frustration Level	Mental Demand	Performance	Physical Demand	Temporal Demand
90	90	90	30	50	100

Sources of Workload tally (number of times selected)

Effort	Frustration Level	Mental Demand	Performance	Physical Demand	Temporal Demand
2	3	3	3	0	4

Adjusted Rating (Weight x Raw)

Effort	Frustration Level	Mental Demand	Performance	Physical Demand	Temporal Demand
180	270	270	90	0	400
